# Supplementary material for: Effects of Botulinum Toxin Therapy on Health-Related Quality of Life Evaluated by the Oromandibular Dystonia Rating Scale
Source: Toxins (Basel). 2022 Sep 22;14(10):656. doi: 10.3390/toxins14100656 (PMC9609031; doi:10.3390/toxins14100656)
Supplement: Supplementary file 1 [file toxins-14-00656-s001.zip › toxins-1911205-supplementary.pdf]

Oromandibular Dystonia Rating Scale (OMDRS)

Examiner-rated Scale

Patient or ID:______________________

Examiner:__________________________

Date examined:_____________________

Video rater:________________________

Date rated:_________________________

1. SEVERITY SCALE

Before rating, the examiner should measure maximum mouth opening, protrusion, and lateral movements. Moreover, protrusion or deviation of the tongue or lip was measured according to the subtypes of the oromandibular dystonia.

The examiner should induce dystonia by requesting the patients to speak or chew according to the video examination protocol and subsequently rate the deviation.

With respect to A (involuntary mouth closing), the examiner should request that the patient close the mouth maximally and forcefully and then ask the following question: “What is the percent force when you close mouth involuntarily compared to the maximum bite force you have just tried?”

A. Involuntary mouth closing.

Absent	0
Slight: less than 25% of the maximum occlusal force	1
Mild: 25 to less than 50% of the maximum occlusal force	2
Moderate: 50 to less than75% of the maximum occlusal force	3
Severe: 75% or more of the maximum occlusal force	4

B. Involuntary mouth opening, deviation or protrusion

Absent	0
Slight: less than 25% of the maximum mouth opening, deviation or protrusion	1
Mild: 25 to less than 50% of the maximum opening, deviation or protrusion	2
Moderate: 50 to less than75% of the maximum opening, deviation or protrusion	3
Severe: 75% or more of the maximum opening, deviation or protrusion	4

C. Involuntary tongue tension

Absent	0
Slight: less than 25% of the maximum protrusion, retraction or deviation	1
Mild: 25 to less than 50% of the maximum protrusion, retraction or deviation	2
Moderate: 50 to less than 75% of the maximum protrusion, retraction or deviation	3

Severe: 75% or more of the maximum protrusion, retraction or deviation	4

D. Involuntary lips and peri-mouth tension

Absent	0
Slight: less than 25% of the maximum deviation	1
Mild: 25 to less than 50% of the maximum deviation	2
Moderate: 50 to less than 75% of the maximum deviation	3
Severe: 75% or more of the maximum deviation	4

E. Duration of involuntary contraction

Absent	0
Occasional: less than 25% of the time	1
Intermittent: 25 to less than 50% of the time	2
Frequent: 50 to less than 75% of the time	3
Constant: 75% more time	4

F. Maximal mouth opening

Mouth opening: 40 mm or more	0
Mouth opening: 25 mm to less than 40 mm	1
Mouth opening: 15 mm to less than 25 mm	2
Mouth opening: less than 15 mm	3
Mouth opening: 0 mm	4

**Total Severity Score**		
Jaw closing dystonia	Sum of A, D, E, F (0-16)	
Tongue dystonia	Sum of B, C, D, E (0-16)	
Jaw opening dystonia	Sum of B, C, D, E (0-16)	
Jaw deviation (protrusion) dystonia	Sum of A, B, E, F (0-16)	
Lip dystonia	Sum of B, D, E, F (0-16)	

2. DISABILITY SCALE

A. Work (occupation or housework/home management).

No difficulty	0
Normal work expectations with satisfactory performance at usual level of occupation but some interference by symptoms	1
Most activities unlimited, selected activities very difficult and hampered but still possible with	2

satisfactory performance	
Working at lower than usual occupational level; most activities hampered, all possible but less than satisfactory performance in some activities.	3
Unable to engage in voluntary or gainful employment; still able to perform some domestic responsibilities satisfactorily	4
Marginal or no ability to perform domestic responsibilities	5

B. Activities of daily living [e.g., feeding, dressing, hygiene (including washing, shaving, makeup, etc.)]

No difficulty with any activity	0
Activities unlimited, but some interference by symptoms	1
Most activities unlimited, selected activities very difficult and hampered but still possible using simple tricks	2
Most activities hampered or laborious but still possible; may use extreme "tricks"	3
All activities impaired; some impossible or require assistance	4
Dependent on others in most self-care tasks	5

C. Activities outside the home (e.g., shopping, walking about, movies, dining or other recreational activities

No difficulty	0
Unlimited activities but bothered by symptoms	1
Unlimited activities but requires use of "tricks" to accomplish	2
Only accomplishes activities when accompanied by others because of symptoms	3
Limited activities outside the home; certain activities impossible or given up due to symptoms	4
Rarely if ever engages in activities outside the home	5

D. Eating

No difficulty	0
Able to eat anything, but it takes a long time	1
Only able to eat soft food	2
Finds it difficult and takes a long time to eat soft food	3
Only able to consume liquids	4
Ingestion impossible	5

E. Swallowing

No difficulty	0
Discomfort during swallowing	1
Swallowing is slightly difficult, but not choking	2
Difficulty in swallowing and may cause choking	3
Difficulty in swallowing, often choking	4
Ingestion impossible	5

F. Speech

No difficulty	0
Speech is not clear, but it does not cause others to ask me to repeat myself.	1
Speech causes people to ask me to occasionally repeat myself, but not every day.	2
Speech is so unclear enough that others ask me to repeat myself every day even though most of my speech is understood.	3
Most of speech cannot be understood.	4
Speech cannot be understood.	5

**Total Disability Score**	
Sum of A, B, C, D, E, F (0-30)	

3. PAIN SCALE

3.1. Rate the severity of pain during the last week on a scale of 0 - 10, where a score of 1 represents a minimal ache and 10 represents the most excruciating pain imaginable

Best (0 to 10)	
Worst (0 to 10)	
Usual (0 to 10)	

3.2. Rate the duration of pain

None	0
Present < 10% of the time	1
Present 10% to < 25% of the time	2
Present 25% to < 50% of the time	3
Present 50% to < 75% of the time	4
Present ≥ 75% of the time	5

3.3. Rate the degree to which pain contributes to disability

No limitation or interference from pain	0
Pain is quite bothersome but not a source of disability	1
Pain definitely interferes with some tasks a major contributor to disability	2
Pain accounts for some (less than half) disability	3
Pain is a major source of difficulty with activities; separate from this, muscle contraction is also a source of some (less than half) disability	4
Pain is the major source of disability; without it, most impaired activities could be performed quite satisfactorily	5

**Total Pain Score**	
Sum of 1, 2, 3 (0-40)	

**Total Examiner-rated Scale Score**	
Sum of Severity, Disability, and Pain (0-86)	

Patient-administered Questionnaire

Because of your symptoms, how often have you experienced the following problems in the past two weeks? Please mark one answer per question.

General (5)

1. How often has your symptoms affected your everyday life?

Never	Seldom	Sometimes	Often	Always
☐	☐	☐	☐	☐

2. Has your work or housework been affected by the symptoms?

Never	Seldom	Sometimes	Often	Always
☐	☐	☐	☐	☐

3. Has it been difficult for you to keep up with the demands of your job or home life?

Not at all	Slightly	Moderately	Severely	Very severely
☐	☐	☐	☐	☐

4. Has it been difficult for you to do things you like to do?

Never	Seldom	Sometimes	Often	Always
☐	☐	☐	☐	☐

5. Has it been difficult for you to control the symptoms when you were nervous or under stress?

Never	Seldom	Sometimes	Often	Always
☐	☐	☐	☐	☐

Eating (7)

1. Has it been difficult for you to chew food?

Never	Seldom	Sometimes	Often	Always
☐	☐	☐	☐	☐

2. Has it been difficult for you to swallow food?

Never	Seldom	Sometimes	Often	Always
☐	☐	☐	☐	☐

3. Has it been difficult for you to keep food in your mouth while chewing or swallowing?

Never	Seldom	Sometimes	Often	Always

□ □ □ □ □

4. Have you injured your tongue, lips or cheeks while eating?

Never Seldom Sometimes Often Always

□ □ □ □ □

5. How often have you had to drink to enable swallowing?

Never Seldom Sometimes Often Always

□ □ □ □ □

6. How often have you dropped food or a liquid while eating?

Never Seldom Sometimes Often Always

□ □ □ □ □

7. Have you had to watch the consistency of the food before eating it?

Never Seldom Sometimes Often Always

□ □ □ □ □

Speech (4)

1. Has it been difficult for you to speak?

Never Seldom Sometimes Often Always

□ □ □ □ □

2. Have you had problems getting understood when talking on the telephone?

Never Seldom Sometimes Often Always

□ □ □ □ □

3. Have you had problems getting understood when in quiet surroundings?

Never Seldom Sometimes Often Always

□ □ □ □ □

4. Have you had problems getting understood when talking in public or in loud surroundings?

Never Seldom Sometimes Often Always

□ □ □ □ □

Cosmetic (7)

1. How often has your symptoms affected your physical appearance?

Never	Seldom	Sometimes	Often	Always
☐	☐	☐	☐	☐

2. Have you felt embarrassed or ashamed because of your look or physical appearance?

Never	Seldom	Sometimes	Often	Always
☐	☐	☐	☐	☐

3. Have you felt the need to conceal your symptoms from other people?

Never	Seldom	Sometimes	Often	Always
☐	☐	☐	☐	☐

4. Have you worried about how other people react to you?

Never	Seldom	Sometimes	Often	Always
☐	☐	☐	☐	☐

5. Have you felt you didn’t look so good?

Never	Seldom	Sometimes	Often	Always
☐	☐	☐	☐	☐

6. Have you preferred to eat alone because of your symptoms?

Never	Seldom	Sometimes	Often	Always
☐	☐	☐	☐	☐

7. Have you avoided going out for dinners?

Never	Seldom	Sometimes	Often	Always
☐	☐	☐	☐	☐

Social/family life (5)

1. Have you felt lonely or isolated because of your symptoms?

Never	Seldom	Sometimes	Often	Always
☐	☐	☐	☐	☐

2. Have you had problems with close friends or your family due to your symptoms?

Never	Seldom	Sometimes	Often	Always
☐	☐	☐	☐	☐

3. Have you avoided situations where many people were present?

Never	Seldom	Sometimes	Often	Always
☐	☐	☐	☐	☐

4. Have you felt unsure or tense with new people?

Never	Seldom	Sometimes	Often	Always
☐	☐	☐	☐	☐

5. Has your symptoms had a negative effect on your family life?

Not at all	Slightly	Moderately	Severely	Very severely
☐	☐	☐	☐	☐

During the past 2 weeks, how often did you:

	None of the time	A little of the time	Some of the time	Most of the time	All of the time
a）Have trouble falling asleep because of the symptoms?	☐	☐	☐	☐	☐
b）Have a restless sleep because of the symptoms?	☐	☐	☐	☐	☐
c）Wake up because of the symptoms?	☐	☐	☐	☐	☐
d）Not getting enough sleep that you needed because of the symptoms?	☐	☐	☐	☐	☐

During the past 2 weeks, has your symptoms limited your ability to carry out your usual social activities?

	Not at all	A little	Moderately	Quite a bit	Extremely
a）Enjoyment of social situations?	☐	☐	☐	☐	☐
b）Socializing with friends of family?	☐	☐	☐	☐	☐

During the past 2 weeks, how often has your symptoms caused you to feel:

	None of the time	A little of the time	Some of the time	Most of the time	All of the time

a）Angry?	◻	◻	◻	◻	◻
b）Annoyed?	◻	◻	◻	◻	◻
c）Irritated?	◻	◻	◻	◻	◻
d）Aggravated?	◻	◻	◻	◻	◻
e）Fed up?	◻	◻	◻	◻	◻
f）Frustrated?	◻	◻	◻	◻	◻
g）Stressed?	◻	◻	◻	◻	◻
h）Impatient?	◻	◻	◻	◻	◻
i）Upset?	◻	◻	◻	◻	◻
j）Worried?	◻	◻	◻	◻	◻
k）Anxious?	◻	◻	◻	◻	◻
l）Scared?	◻	◻	◻	◻	◻
m）Fearful?	◻	◻	◻	◻	◻
n）Depressed?	◻	◻	◻	◻	◻
o）Down?	◻	◻	◻	◻	◻
p）More self-conscious in social situations?	◻	◻	◻	◻	◻
q）Uneasy talking to strangers?	◻	◻	◻	◻	◻
r）Less relaxed in social situations?	◻	◻	◻	◻	◻
s）Embarrassed about eating in public (e.g. café, restaurant)?	◻	◻	◻	◻	◻
t）Embarrassed going out in public (ego cinema, theater)?	◻	◻	◻	◻	◻
u）Everybody is staring at you?	◻	◻	◻	◻	◻
v）Lack of confidence?	◻	◻	◻	◻	◻
w）Lack of self-confidence?	◻	◻	◻	◻	◻

**Total Patient-rated Questionnaire Score** (0-228)	

**Total OMDRS Score**	
Sum of total Examiner-rated Scale Score and	

total Patient-rated Questionnaire Score (0-314)	
